# Adjuvant Cannabinoid Receptor Type 2 Agonist Modulates the Polarization of Microglia Towards a Non-Inflammatory Phenotype in Experimental Pneumococcal Meningitis

**DOI:** 10.3389/fcimb.2020.588195

**Published:** 2020-11-05

**Authors:** Steven D. Pan, Denis Grandgirard, Stephen L. Leib

**Affiliations:** Neuroinfection Laboratory, Institute for Infectious Diseases, University of Bern, Bern, Switzerland

**Keywords:** bacterial meningitis, microglia, endocannabinoid system, neuro-inflammation, brain damage

## Abstract

**Background:**

Microglia initiates and sustains the inflammatory reaction that drives the pathogenesis of pneumococcal meningitis. The expression of the G-protein cannabinoid receptor type 2 (CB2) in the brain is low, but is upregulated in glial cells during infection. Its activation down-regulates pro-inflammatory processes, driving microglia towards an anti-inflammatory phenotype. CB2 agonists are therefore therapeutic candidates in inflammatory conditions like pneumococcal meningitis. We evaluated the effects of JWH-133, a specific CB2 agonist on microglial cells, inflammation, and damage driven by *S. pneumoniae* *in vitro* and in experimental pneumococcal meningitis.

**Materials/methods:**

Primary mixed glial cultures were stimulated with live or heat-inactivated *S. pneumoniae*, or lipopolysaccharide and treated with JWH-133 or vehicle. Nitric oxide and cytokines levels were measured in the supernatant. *In vivo*, pneumococcal meningitis was induced by intracisternal injection of live *S. pneumoniae* in 11 days old Wistar rats. Animals were treated with antibiotics (Ceftriaxone, 100 mg/kg, s.c. bid) and JWH-133 (1 mg/kg, i.p. daily) or vehicle (10% Ethanol in saline, 100 µl/25g body weight) at 18 h after infection. Brains were harvested at 24 and 42 h post infection (hpi) for histological assessment of hippocampal apoptosis and cortical damage and determination of cyto/chemokines in tissue homogenates. Microglia were characterized using Iba-1 immunostaining. Inflammation in brain homogenates was determined using membrane-based antibody arrays.

**Results:**

*In vitro*, nitric oxide and cytokines levels were significantly lowered by JWH-133 treatment. *In vivo*, clinical parameters were not affected by the treatment. JWH-133 significantly lowered microglia activation assessed by quantification of cell process length and endpoints per microglia. Animals treated with JWH-133 demonstrated significantly lower parenchymal levels of chemokines (CINC-1, CINC-2α/β, and MIP-3α), TIMP-1, and IL-6 at 24 hpi, and CINC-1, MIP-1α, and IL-1α at 42 hpi. Quantitative analysis of brain damage did not reveal an effect of JWH-133.

**Conclusions:**

JWH-133 attenuates microglial activation and downregulates the concentrations of pro-inflammatory mediators in pneumococcal infection *in vitro* and *in vivo*. However, we didn’t observe a reduction in cortical or hippocampal injury. This data provides evidence that inhibition of microglia by adjuvant CB2 agonists therapy effectively downmodulates neuroinflammation but does not reduce brain damage in experimental pneumococcal meningitis

## Introduction

In bacterial meningitis an overshooting inflammatory reaction in the central nervous system contributes to the pathophysiology of the disease including the development of brain damage. Specifically, pneumococcal meningitis is characterized by a high rate of mortality and morbidity, even when patients are treated with efficient antibiotic therapy. Survivors, in particular children, are left with several long-lasting disabilities, the most frequent being hearing loss, but also cognitive impairments, including learning and memory deficits, as well as focal neurological deficits (Edmond et al., 2010; Agyeman et al., 2014; Lucas et al., 2016; Muri et al., 2019a). The causes of these different sequelae have been deduced from the histological analyses of tissues of deceased patients or from experimental models. Hearing loss has been related to damage in the inner ear, including loss of hair cells or spiral ganglion neurons and from cochlear ossification (Klein et al., 2003; Perny et al., 2016). Focal neurological deficits are mostly caused by cerebrovascular events or intracerebral bleeding and characterized by the occurrence of cerebral infarcts due to localized hypoxia/ischemia or hemorrhages, respectively (Ment et al., 1986; Auer et al., 2000; Vergouwen et al., 2010). Finally, the development of cognitive impairments was linked to the detection of hippocampal damage, including apoptosis in the dentate gyrus (Nau et al., 1999; Wellmer et al., 2000; Loeffler et al., 2001; Leib et al., 2003; Grandgirard et al., 2007a).

On the pathophysiological level, these damages are the consequence of an intensive inflammatory reaction initiated by the recognition of bacterial compounds by endothelial cells, microglia and perivascular macrophages. The further recruitment of neutrophils in the cerebrospinal fluid contributes to the over-production of pro-inflammatory mediators, including cytokines, chemokines, matrix-metalloproteinases, and nitric oxide. Together with the release of bacterial toxins, they contribute directly or indirectly to the development of the different forms of damage described above (Mook-Kanamori et al., 2011; Agyeman et al., 2014).

Limiting this detrimental inflammatory reaction has therefore been extensively investigated (Van Der Flier et al., 2003; Grandgirard and Leib, 2006; Bewersdorf et al., 2018), but to date, adjuvant corticosteroids is the only recommended therapeutic option (Van De Beek et al., 2016). It has been proven beneficial, in specific patient populations, especially in high income countries, for the prevention of hearing loss (Brouwer et al., 2015). However, detrimental effects of dexamethasone therapy has been observed in a number of experimental models (Leib et al., 2003; Spreer et al., 2006; Bally et al., 2016) and dexamethasone may predispose patients to delayed cerebral thrombosis (Lucas et al., 2013).

Thus, alternative anti-inflammatory approaches for the adjuvant therapy of BM are being evaluated. Recently, the endocannabinoid system has received considerable attention for its potential to modulate inflammation and pain disorders. Immune cells, regardless of their lineage, express cannabinoid receptors (CB). These receptors are divided into multiple subtypes, the most common being central cannabinoid receptor type 1 (CB1) and peripheral cannabinoid receptor type 2 (CB2) (Facci et al., 1995; Mccoy, 2016). CB1 is primarily expressed in the CNS and various peripheral tissues. On the other hand, CB2 is prevalent within all lineage of the immune system. In the healthy brain, CB2 expression is limited (Svizenska et al., 2008; Turcotte et al., 2016). However, during neurological diseases, glial cells express high levels of CB2 (Ashton and Glass, 2007; Stella, 2010). *In vitro* experiments demonstrated that activation of CB2 by its endogenous ligands, the endocannabinoids, led to contrasting results. While activation with 2-arachidonoyl-glycerol (2-AG) mostly up-regulated functions related to leukocytes recruitment, *N*-Arachidonoyl-ethanolamide (AEA) down-regulated leukocyte functions, such as pro-inflammatory cytokine release and nitric oxide production (Turcotte et al., 2016). In contrast to endocannabinoids, exogenous CB2 receptor agonists exert exclusively anti-inflammatory activity. It has been for example demonstrated that JWH-015 repressed LPS-induced TNF-α production and migration in microglial cells (Romero-Sandoval et al., 2009). It also suppressed TNF-α and nitric oxide production induced by IFN-γ or Aβ peptide (Ehrhart et al., 2005). Furthermore, *in vivo* studies demonstrated that CB2 knockout mice were characterized by the development of an exacerbated inflammatory response, including increased leukocyte recruitment and pro-inflammatory cytokine production, which often caused tissue damage. In particular, CB2 -/- mice with traumatic brain injury displayed an increased gene expression of TNF-α, iNOS and ICAM, accompanied by an elevated blood brain barrier permeability (Amenta et al., 2014).

JWH-133 is a potent and selective CB2 agonist, with no activity on CB1 receptor, in both human and mouse. It has therefore been recommended as one of the most suitable CB2 agonist for preclinical target validation (Soethoudt et al., 2017). It has already been shown to have beneficial effects, including downregulation of the inflammation and the improvement in neurofunctional outcome in different experimental models of brain injury, including okadaic-induced neurodegeneration (Cakir et al., 2019), subarachnoid hemorrhages (Fujii et al., 2014a; Fujii et al., 2014b), stroke (Zarruk et al., 2012; Li et al., 2015; Bravo-Ferrer et al., 2017), endotoxemia (Gamal et al., 2015), traumatic brain injury (Amenta et al., 2014) or Parkinson’s disease (Chung et al., 2016).

Since pneumococcal meningitis shares pathophysiological mechanisms with some of the brain diseases successfully targeted by JWH-133, we hypothesized that its application as adjuvant therapy may prevent the excessive neuroinflammation by reducing pro-inflammatory glial activity during the acute phase of the disease and attenuate brain damage.

## Materials and Methods

### Infecting Organism

A clinical isolate of *Streptococcus pneumoniae* (serotype 3) from a patient with bacterial meningitis was cultured overnight in Brain Heart Infusion (BHI) medium, diluted tenfold in pre-warmed BHI, and grown for 5 h to reach logarithmic growth phase. Bacteria were then centrifuged for 10 min at 3,100 × g and resuspended in 0.85% saline (NaCl). After a second wash in saline, bacteria were further diluted with saline to the desired optical density at 570 nm, so to obtain a mean concentration of approx. 1 × 10^7^ CFU/ml. Inoculum concentration was determined using serial dilution and plating on Columbia sheep blood agar (CSBA) plates.

### *In Vitro* Mixed Glia Stimulation

Mixed glial cultures consisting of microglial and astroglial cells were isolated from infant rat brains at postnatal day 3 (P3) as previously described (Muri et al., 2019b). Cortex was mechanically homogenized in PBS by pipetting up and down, and resuspended in DMEM (Sigma-Aldrich, Merk Switzerland) containing 5% FCS (Biochrom, Germany), 1% GlutaMAX^TM^ (ThermoFisher, Switzerland) and antibiotic-antimycotic solution (100 units/ml penicillin and streptomycin, 0,25 µg/ml Amphotericin B, Thermofisher, Switzerland) and plated in a poly-L-ornithine-coated T75 flask. On day 11, cells were seeded at a density of 200,000 cells/well onto a poly-o-ornithine coated 24-well plate. Mixed glial cells were challenged with 1 μg/ml of lipopolysaccharide (*Escherichia coli*, L2654, Sigma-Aldrich), live *S*. *pneumoniae* serotype 3 (9.1 × 10^8^ CFU/ml) in presence of Ceftriaxone (CRO, 12 mg/ml, Rocephine, Roche) or PBS as a control. Each group was treated with 1.0 μM of JWH-133 (Tocris Bioscience) compared to untreated cells. Three independent experiments were performed with all conditions in triplicates.

### Quantification of Nitric Oxide Production From Mixed Glial Cell Stimulation

After 42 h challenge, 100 μl of cell culture supernatant was mixed with 100 μl of Griess reagent (Sigma-Aldrich) in a 96-well plate. NO_2_- concentration, serving as an indicator of NO release, was determined by measuring absorbance at 550 nm with a microplate reader (Molecular Devices, THERMO max). A serial dilution of NaNO_2_ from 100–1.625 μM was used to generate a standard curve. All measures were performed in triplicate and a mean value was calculated

### Quantification of Cyto/Chemokines in the Supernatants of Stimulated Mixed Glial Cells

Cytokines known to be upregulated during PM (IL-1β, IL-6, TNF-α, IL-10, and IFN-γ) were assessed using magnetic multiplex assay (Rat Magnetic Luminex® Assay, Rat Premixed Multi-Analyte Kit, R&D Systems, Bio-Techne) on a Bio-Plex 200 station (Bio-Rad Laboratories) as described previously (Muri et al., 2019a). Fifty µl of cell culture medium was used undiluted. For each sample, a minimum of 50 beads was measured. If the concentration of the sample was below the detection limit, a value corresponding to the detection limit provided by the manufacturer was used, considering the dilution factor. The detection limits for undiluted samples were 2.93 pg/ml for IL-1β, 23.2 pg/ml for IL-6, 8.95 pg/ml for IL-10, 11.5 pg/ml for TNF-α, and 70.9 pg/ml for IFN-γ.

### *In Vivo* Pneumococcal Meningitis Model

All animal experiments were approved by the Animal Care and Experimentation Committee of the Canton of Bern (BE 1/18). A well characterized *in vivo* model of pneumococcal meningitis was used (Leib et al., 2001; Perny et al., 2016). Eleven-day old mixed gender Wistar rats and their dams were purchased from Charles River (Sulzfeld, Germany), and housed at room temperature (22 ± 2°C) in natural light. The pups were infected intracisternally with 10 μl of live *S*. *pneumoniae* serotype 3 (1.14 x 10^7^ ± 7.5 x 10^6^ CFU/ml). Control animals were injected with 10 μl of 0.85% NaCl. Meningitis was confirmed through quantification of bacterial titers from cerebrospinal fluid (CSF) harvested from animals at 18 hpi. CSF was harvested through puncture of the cisterna magna with a 30-guage needle and diluted in saline for plating on CSBA plates. Disease symptoms were scored as following: (1) = minimal or no spontaneous motor activity, coma (2) = unable to turn upright (3) = turns upright within 30s (4) = signs of disease in terms of weight loss and/or appearance of fur, (5) = healthy, normal behavior. Spontaneous mortality was documented and animals with a score of 2 or lower were sacrificed.

A total of 84 infant rats were used for this study, representing 7 independent experiments of 12 animals. All animals were sacrificed during the acute phase of pneumococcal meningitis to assess neuroinflammation. Both infected and uninfected animals were randomized for treatment with JWH-133 (1mg/kg, i.p.) and/or CRO (100 mg/kg, i.p). JWH-133 was first dissolved in 100% ethanol and diluted 1:10 in 0.9% saline at a final concentration of 0.25 mg/ml). JWH-133 was administered once at 18 hpi, and CRO was administered at 18 hpi and 24 hpi. Animals not treated with JWH-133 received an equivalent volume (100 µl/25 g) of vehicle. Depending on the endpoints, animals were sacrificed at 24 hpi and 42 hpi with an overdose of pentobarbital (Eskonarkon, Streuli Pharma AG, Uznach, Switzerland, 150mg/kg b.w. i.p) and perfused via the left ventricle with either 4% paraformeldahype (PFA) in PBS or ice-cold PBS.

### Histological Analysis of Cortical Damage and Hippocampus Apoptosis

Brains were harvested at 42 hpi after perfusion of animal with 4% PFA and fixed in 4% PFA for 4 h at 4°C. After 4 h, brains were transferred into 18% sucrose and kept at 4°C overnight. Brain cryosections (45 μM) were stained for Nissl substance with cresyl violet. Cortical damage was quantified using ImageJ software, and apoptosis was measured in the hippocampal dentate gyrus using x 400 magnification.

### Iba1 Staining of Microglial Cells

Brains harvested at 42 hpi were sampled into 50 μm free-floating cryosections. Microglia immunostaining was performed with ImmPRESS^TM^ HRP anti-rabbit IgG Peroxidase Polymer Detection Kit (Vector Laboratories, USA) in conjunction with rabbit anti Iba-1 (WAKO, Germany). Free-floating sections were incubated at room temperature for 72 h in 1 ml of primary Iba-1 antibody diluted 1:400. Endogenous peroxidase activity was blocked with 3% hydrogen peroxide, followed by incubation for 20 min in 2.5% normal goat blocking serum. Sections were then incubated in the ImmPRESS^TM^ anti-rabbit peroxidase polymer for 30 min. Microglia were visualized following a 2-minute incubation with Vector^©^ 3,3’-diaminobenzidine (DAB) substrate (Vector Laboratories, USA).

### Quantification and Categorical Analysis of Microglia Morphology

Quantification was performed in a subset of the animals, randomly chosen in each experimental group with Iba-1 staining of sufficient good quality. Our method of microglia quantification was adapted from a previously described methodology (Young and Morrison, 2018). Nine separate images of microglia in the cortex and hippocampus of 50 μm brain sections were randomly sampled under x 400 magnification from each animal (3 images per section, 3 sections per animal). Through ImageJ, microglia images were passed through an unsharp mask filter and converted into an 8-bit image, and then a binary image. Incomplete microglia structures around the periphery of the image were cleared. The binary image of microglia was then skeletonized with the AnalyzeSkeleton plugin, which allowed to measurement of cell branching and summed branch length. The summed endpoints and branch length were divided by the number of cell bodies in the visual field to determine the endpoints/cell and branch length/cell of the microglia visualized.

Using a previously described characterizations of microglia activations states (Davis et al., 1994; Davis et al., 2017), cells were classified into three categories: resting = round, oval body with thin, long and radially projecting processes; intermediate = enlarged and darkened cell bodies with thick processes and less branching; active = enlarged, darkened cell bodies with little to no processes observed.

### Quantification of Cyto/Chemokines in the Brain Parenchmya

Quantification of neuroinflammation in the parenchyma was performed using a membrane-based immunoassay containing a panel detecting 29 inflammatory parameters (Proteome Profiler Rat Cytokine Array Panel A, R&D Biosystems, Biotechne). Brains were harvested from animals perfused with ice cold PBS at 24 and 42 hpi. The brains were snap frozen on dry ice and stored at -80°C until further processing. For preparation of homogenates, brain samples were thawed on ice, and a portion of the frontal cortex was excised. Samples were homogenized in a 7-ml glass tissue grinder (Kontes Glass Co., USA) with a 7× volume to mass ratio in a buffer consisting of ice-cold PBS, 1% Triton-X-100, and protease inhibitors (cOmplete™, Mini, EDTA-Free, Sigma-Adrich, Merk, Switzerland). The homogenate was cleared by centrifugation at 16000 × g, for 10 min at 4°C. Protein concentration was determined using Pierce™ BCA Protein Assay kit (ThermoFischer Scientific). Each membrane was incubated with 900μg of protein extract and processed according to the manufacturer’s instructions. Enhanced chemiluminescent detection (ECL) was performed on a Fusion FX-6 imaging system (Vilber Lourmat, Marne-la-Vallée, France). Spot density was determined on digitalized images using Image J for the analysis (V. 1.45, National Institutes of Health, Bethesda, Maryland, US) and normalized using internal controls integrated on each membrane.

### Statistical Analysis

All statistical analyses were performed with GraphPad Prism (Prism 8; GraphPad Software Inc., San Diego, USA). Results are presented as mean values ± standard deviation if not stated otherwise. Survival was calculated using a log rank (Mantel-Cox) test. To compare differences between two groups, an unpaired Student t test or a non-parametric Mann-Whitney test were used. To compared difference between multiple groups, we use one-way ANOVA with Tukey’s multiple comparison test. For combined *in vitro* experiments, we performed a repeated measure two-way ANOVA with Sidak multiple comparison for NO and multiple t test with Holm Sidak multiple comparison for cytokines. For clinical scores and weight changes, we used a repeated measure mixed effect model (because of missing values at later time points). A value of p < 0.05 was considered as significant.

## Results

### JWH-133 Reduces Inflammatory Cytokine Levels and Nitric Oxide Production *In Vitro*

**Figure 1:**
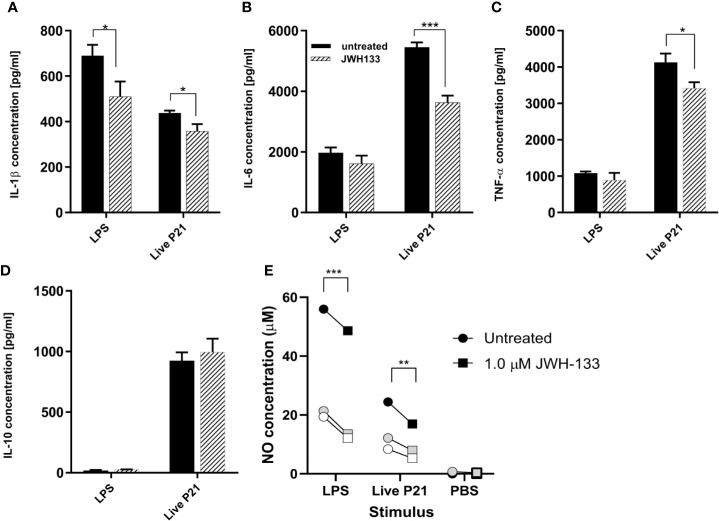
*In vitro*, JWH-133 treatment attenuates LPS or *S. pneumoniae*-induced activation of glial cells. The expression of IL-1β **(A)**, IL-6 **(B)**, and TNF-α **(C)** was reduced by JWH-133 during challenge of astroglial cells by *S. pneumoniae*. JWH-11 also attenuated LPS-induced production of IL-β, but had not effect on IL-10 **(D)**. Nitric oxide production **(E)** upon LPS- and *S. pneumoniae* activation was reduced by JWH-133. (For cytokines, n = 3 for each group; for NO: three independent experiments represented by black, gray, and white symbols; *p < 0.05; **p < 0.01, ***p < 0.001).

Following stimulation of mixed glial cells with LPS, *S*. *pneumoniae* serotype 3, and heat inactivated *S*. *pneumoniae*, each stimulation condition was immediately treated with JWH-133. Both NO and cytokine concentrations showed that LPS and live bacteria induced inflammatory responses in the mixed glial cells (**Figures 1A–E**). In cells stimulated with LPS, JWH-133 significantly decreased NO concentration (p < 0.0001), as well as IL-1β concentration (p = 0.019). In cells stimulated with live *S*. *pneumoniae* serotype 3, JWH-133 significantly decreased concentrations of NO (p = 0.0071), IL-6 (p = 0.00034), IL-1β (p = 0.014), and TNF-α (p = 0.014). Challenge with LPS didn’t efficiently stimulated the production of IL-10, and stimulation with live bacteria wasn’t affected by JWH-133 treatment. In cells stimulated with PBS, NO levels were observed in very low concentrations and cytokines levels were under detection limit. No significant difference between treated and untreated cells were observed in cells stimulated with PBS only.

### Clinical Parameters of Animals With Pneumococcal Meningitis Are Not Impacted by JWH-133 Administration

A total of 84 infant rats were used in this study, of which 70 were infected with *S. pneumoniae*. The development of productive bacterial meningitis was proven in all 70 animals, with CSF bacterial titers ≥ 10^6^ CFU/ml and clinical scores inferior to 5, both determined at 18 hpi. Of these infected animals, 35 were concomitantly treated with JWH-133 and CRO, while the remaining were treated with CRO only.

**Figure 2:**
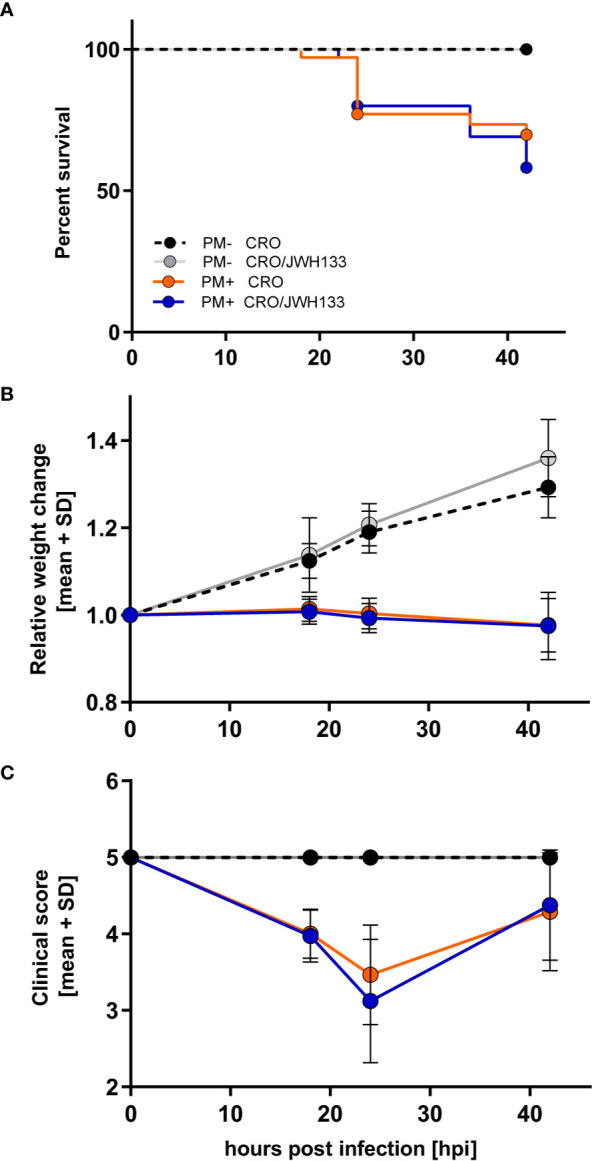
Survival and time-course of clinical parameters during experimental pneumococcal meningitis. No differences could be observed in survival **(A)**, relative weight changes **(B)** or clinical score **(C)** between animals treated with ceftriaxone (CRO) or ceftriaxone combined to adjuvant JWH-133 (CRO/JWH133), neither in uninfected (PM-) nor infected (PM+) animals (PM- CRO and PM- CRO/JHW-133, n = 7; PM+ CRO and PM+ CRO/JWH133, n = 19–35, depending on the time points).

Survival, relative weight change, and clinical scores were significantly different in animals infected with PM compared to uninfected animals. However, in infected animals, adjuvant JWH-133 treatment did not alter these clinical parameters when compared to animals treated with CRO only (**Figures 2A–C**).

### Microglia Morphology and Distribution Are Affected by JWH-133 Administration

A prominent characteristic of microglia is their different branching and cell body morphology linked to their activation states (Davis et al., 1994; Karperien et al., 2013; Gomez-Nicola and Perry, 2015). To assess how JWH-133 attenuates inflammation through microglia modulation, the morphology and activation of microglia cells were quantified on Iba-1 immuno-stained sections.

#### Quantitative Assessment of Branching and Cell Process Length

**Figure 3:**
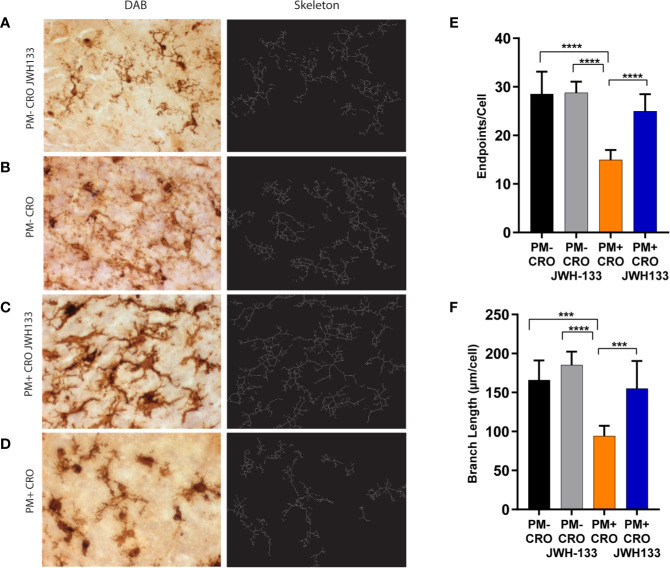
Quantitative phenotypic analysis of microglial activation state on Iba-1 immuno-stained sections. Brain sections of animals from each experimental group, were immuno-stained for microglia using Iba-1 (pictures on the left, **A**–**D**). The digital image was skeletonized (pictures on the right, **(A**–**D)** and endpoints/cell **(E)** or summed branch length **(F)** were determined. In infected animals with ceftriaxone treatment (PM+ CRO), both parameters were significantly reduced compared to the others groups. In contrast, no differences between infected animals treated with JWH-133 (PM+ CRO/JWH133) and uninfected animals (PM-) could be observed. (PM- CRO, n = 3; PM- CRO/JWH133, n = 5; PM+ CRO, n = 11; PM+ CRO/JWH133, n = 6; one-way ANOVA with Tukey’s multiple comparison test: ***p < 0.001; ****p < 0.0001).

The microglia images were skeletonized on ImageJ software, as previously described in the methods (**Figures 3A–D**). These images were analyzed for the endpoints per cell body, as well as the total summed branch length per cell body. Infected animals treated with CRO exhibited significantly fewer endpoints per cell as well as branch length when compared with every other experimental groups. In particular, the difference between infected animals that received JWH-133 or not was highly significant for endpoints (p < 0.0001) and branch length (p < 0.001). In contrast, infected animals treated with JWH-133 didn’t exhibit differences in branch length and endpoints when compared to uninfected animals (**Figures 3E, F**).

#### Classification of Microglia in Three Categories Based On Morphology

**Figure 4:**
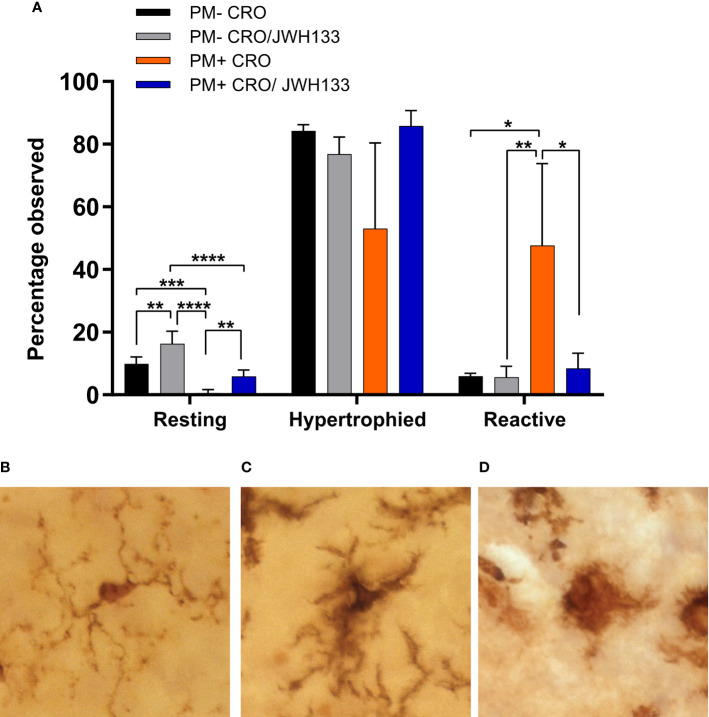
Classification of microglia based on their morphology on Iba-1 immuno-stained sections. Significance differences in the proportion of the microglia classified in the categories “resting” and “reactive” were found between the experimental groups **(A)**. Examples of microglia classified into the three different categories: resting = round, oval body with thin, long, and radially projecting processes **(B)**; hypertrophied = enlarged and darkened cell bodies with thick processes and less branching **(C)**; reactive = enlarged, darkened cell bodies with little to no processes observed **(D)**. PM- CRO, n = 3; PM- CRO/JWH133, n = 4; PM+ CRO, n = 8; PM+ CRO/JWH133, n = 4; one-way ANOVA with Tukey’s multiple comparison test: *p < 0.05; **p < 0.01; ***p < 0.001; ****p < 0.0001.

In conjunction with quantitative assessments of microglia morphology, the Iba-1+ cells were also classified in three categories, based on their morphology. In uninfected animals, more microglial cells possessed thin processes with light staining of cell bodies (**Figure 4B**). These characteristics are indicative of “resting” microglia, which patrol the body under physiological conditions (Davis et al., 1994). Interestingly, JHW-133 increased the proportion of this microglial type not only in infected animals, but also in uninfected. In infected animals treated only with CRO, Iba-1 staining showed significantly more cells with little to no processes, with enlarged bodies and an amoeboid shape (**Figure 4D**) compared to animals treated with JHW-133. Between these two phenotypes, microglia can possess thicker processes and larger cell bodies (Davis et al., 1994; Davis et al., 2017). These microglial cells are believed to be an intermediate between ramified and reactive microglia, and have been referred to as “bushy”, “hypertrophied”, “rod-like” and “bipolar” (Davis et al., 2017). In our study, we characterized these cells as “hypertrophied” (**Figure 4C**). In contrast to the treatment with CRO alone, the brain of infected animals with adjuvant JWH-133 exhibited a majority of Iba-1^+^ cells possessing the phenotypic characteristics of hypertrophied microglia (**Figure 4A**).

#### Quantitative Assessment of Microglial Density

The total number of Iba-1+ cells was assessed to determine whether adjuvant JWH-133 influences the density of microglia. No statistically significant difference caused by JWH-133 treatment, neither in uninfected nor in infected animals was observed. Furthermore, when comparing infected and uninfected animals, no significant increase in microglial density was documented (**Supplementary Figure 1**).

### JWH-133 Reduces the Expression of Inflammatory Cyto/Chemokines, But Not of MMP-9 in the Parenchyma of Animals With Pneumococcal Meningitis

**Figure 5:**
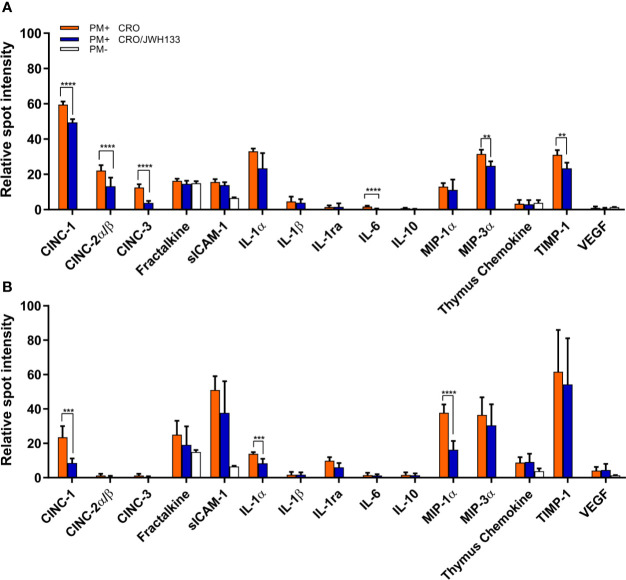
Analysis of inflammatory parameters in the brain parenchyma of infected animals. At 24 hpi **(A)** and 42 hpi **(B)**, the protein levels of several inflammatory parameters were significantly decreased in infected animals treated with CRO/JWH-13 (blue bars) compared to animals treated with CRO only (orange bars). Only few inflammatory parameters could be detected in uninfected animals (white bars). (PM+ CRO, n = 6; PM+ CRO/JWH133, n = 6; PM-, n = 4; Multiple t test with Holm-Sidak correction; **p < 0.01; ***p < 0.001; ****p < 0.0001).

In order to analyze the inflammation in the parenchyma over the course of acute meningitis, brain homogenates of animals sacrificed at 24 and 42 hpi were used to profile an array of inflammatory cytokines. With the exception of fractalkaline, sICAM-1, and thymus chemokine, inflammatory proteins were not detected in uninfected animals, while animals with PM demonstrated detectable levels of several inflammatory cytokines (**Figures 5A, B**). At 24 hpi, the animals treated with JWH-133 demonstrated significantly decreased levels of several neutrophil chemoattractants, including CINC-1 (p < 0.001), CINC-2α/β (p = 0.0037), and CINC-3 (p < 0.001). At 42 h, the only chemoattractant that was significantly reduced was CINC-1 (p < 0.001). Inflammatory interleukin levels were also reduced in the parenchyma after administration of JWH-133 (**Figures 5A, B**). At 24 h, IL-6 was significantly reduced (p < 0.001), and at 42 h, IL-1α was significantly reduced compared to animals treated with only CRO (p < 0.001). Macrophage inflammatory proteins were also significantly reduced following JWH-133 administration at both 24 and 42 hpi (**Figures 5A, B**). Levels of MIP-3α (CCL20) were significantly reduced at 24 hpi (p < 0.001), and levels of MIP-1α (CCL3) were reduced by JWH-133 at 42hpi (p < 0.001). While JWH-133 administration reduced levels of inflammatory cytokines, it also reduced the level of metalloproteinase inhibitor TIMP-1 at 24 hpi (p = 0.0019). In contrast, we couldn’t detect a reduction in MMP-9, neither at 24 hpi, nor 48 hpi (**Supplementary Figure 2**).

### Adjuvant JWH-133 Doesn’t Significantly Alter Cortical Damage or Hippocampus Apoptosis

**Figure 6:**
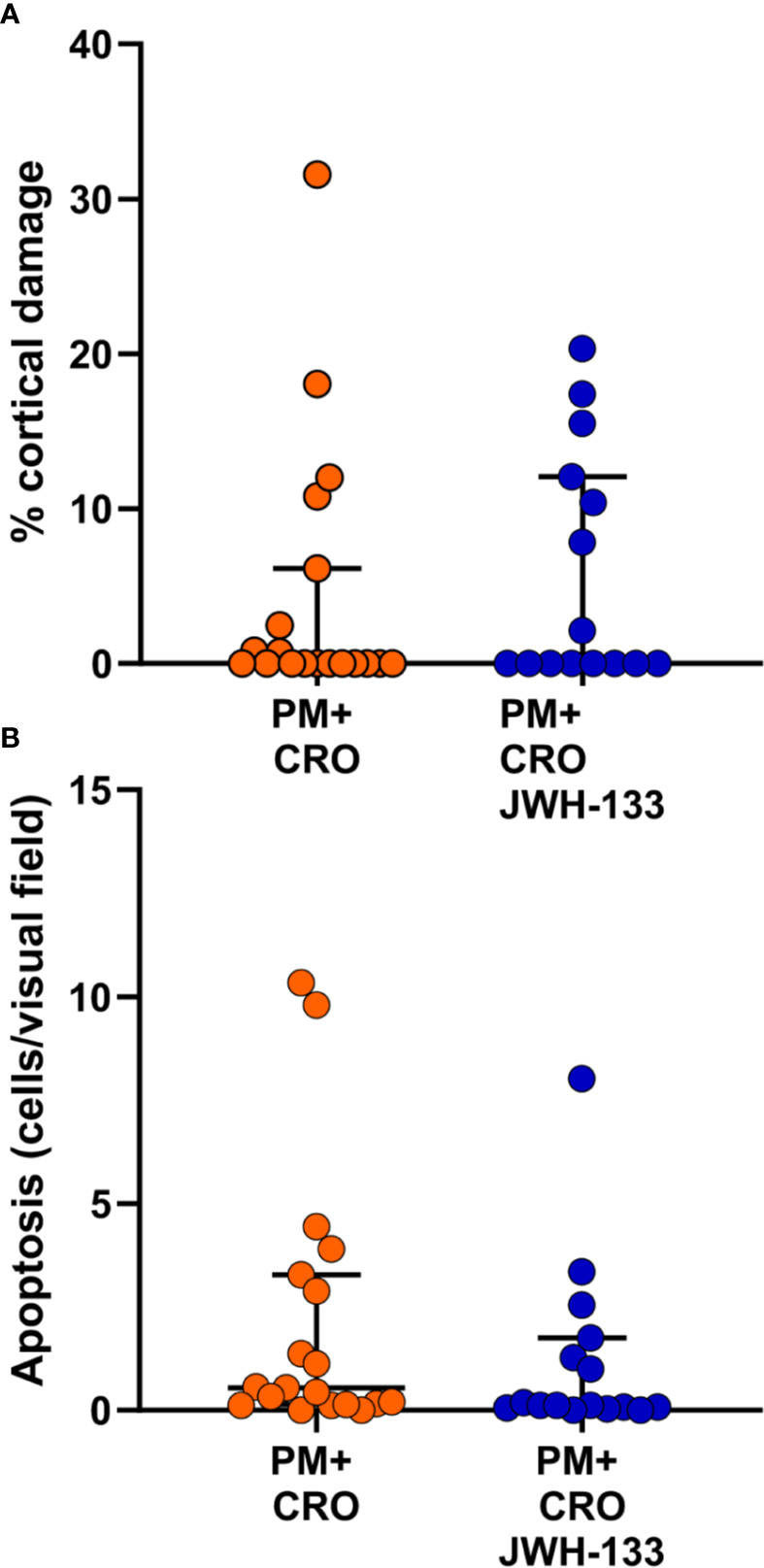
Levels of brain damage. No significant differences for cortical damage **(A)** nor for hippocampal apoptosis **(B)** could be detected between infected animals treated with CRO alone (orange dots) or CRO/JHW133 (blue dots). (PM + CRO, n = 19; PM+ CRO/JWH133, n = 16; Mann-Whitney test: cortical damage, p = 0.62; hippocampal apoptosis, p = 0.13).

To further assess the effects of JWH-133, cortical damage and hippocampal apoptosis were analyzed. No cortical damage was observed in uninfected animals with CRO or CRO/JWH133 administered. In animals infected with PM, administration of JWH-133 didn’t reduce the cortical damage nor hippocampal apoptosis (**Figure 6**).

## Discussion

Pneumococcal meningitis induces inflammation in the CNS, that can be exacerbated by bacteriolytic antibiotics such as CRO, leading to long term neurological sequelae (Mook-Kanamori et al., 2011). While dexamethasone is currently used as an adjuvant therapeutic, its long-term neuroprotective properties on hearing loss and memory impairment are limited (Leib et al., 2003; Van De Beek, 2009) and concerns exist about corticosteroids being responsible of delayed cerebral thrombosis after initial good recovery (Schut et al., 2009; Gallegos et al., 2018). Thus, studying the efficacy of alternative therapeutic candidates for PM is important for the improvement of clinical outcome following infection and the identification of new treatment modalities.

In this study, we assessed the anti-inflammatory and neuroprotective effects of JWH-133 administration. Previous studies have shown the protective properties of administering specific endocannabinoid receptor type 2 agonists in various disease models. JWH-133 has a very high affinity and specificity for CB2 (K_i_ CB2 3.4 nM/K_i_ CB1 680 nM) (Huffman et al., 1999) when compared to other CB2 agonists, like JWH-015 (K_i_ CB2: 13.8 nM/K_i_ CB1: 383 nM) (Pertwee, 1999). Importantly, JWH-015 has been reported to have seven off-target receptors, while JWH-133 had none (Soethoudt et al., 2017). As a consequence, JWH-015 may act independently from CB receptors, for example through the glucocorticoid receptor (Fechtner et al., 2019).

So far, few studies have addressed the use of CB2 agonists in infectious diseases (Hernandez-Cervantes et al., 2017). These studies have mostly used non-selective agonists, including tetrahydrocannabinol (THC), cannabidiol (CBD) or marijuana extracts. For bacterial infection, a model of sepsis using cecal ligation and puncture (CLP) demonstrated that the selective CB2 agonist Gp1, given shortly after CLP induction, decreased neutrophil recruitment, while increasing neutrophil activation at the site of infection. Further, it decreased pulmonary damage (Tschop et al., 2009). Non-selective inhibitors have also been investigated in paradigms of viral infections. A beneficial effect was found in viral infections where inflammation participates in viral spread and is detrimental. For example, CB2 agonists have the potential to reduced HIV-associated neurocognitive disorders (Purohit et al., 2014). JWH-133 was shown to reduce lung inflammation and damage in experimental respiratory syncytial virus infection in mice (Tahamtan et al., 2018). Very recently, therapy using CB2 agonist has been proposed to control the cytokine storm observed during acute cases of SARS-CoV-2 infection (Rossi et al., 2020)

Since CB2 agonists are generally considered as immunosuppressive, the timing of application is critical. Inactivation of CB2 prior to infection or using knockout models may be detrimental. CB2 deficiency may prolong host exposure to pathogens, decrease viral clearance, and broke down immune cell crosstalk, such as neutrophil migration. This way, treatment with CB2 agonists may render individuals more susceptible to infections (Hussain et al., 2019). Evidence for a role of CB2 deficiency as a risk factor for bacterial infection of the CNS is lacking so far. A review paper by Gowin and colleagues (Gowin and Januszkiewicz-Lewandowska, 2018) inventoried SNPs involved in bacterial meningitis, but CB2-related SNPs were not reported.

Here, the efficacy of a very specific CB2 agonist is evaluated for its ability to reduce neuroinflammation during the acute phase of bacterial meningitis, with a special focus on microglia. Our results indicate that JWH-133 exerts anti-inflammatory effects on glial cells *in vitro* and that its administration as adjuvant therapy *in vivo* modulates microglia activity in the CNS, thus also exerting an anti-inflammatory effect.

Our *in vitro* data with mixed glial cell cultures demonstrate anti-inflammatory properties of JWH-133 in both LPS and live *S*. *pneumoniae* serotype 3-stimulated cells. JWH-133 reduced IL-6, IL-1, and TNF-α, all of which have been shown to play an important role in upregulating neuroinflammation in PM (Coutinho et al., 2013). Thus, attenuating the levels of these cytokines may have significant neuroprotective effects in PM, and improve outcomes. We have also shown that NO concentrations were significantly reduced in cultures treated with either LPS or living *S*. *pneumoniae*. NO is a signaling molecule released by macrophages and is involved in inflammatory processes, including vasodilation (Sharma et al., 2007). During infection, microglial cells are a major source of inflammatory cytokines (Hanisch, 2002), including IL-1β, IL-6-α, and TNF-α (Lee et al., 1993; Hanisch, 2002). Thus, the attenuated production of these cytokines and of NO by mixed glial cell cultures *in vitro* suggests that the agonist JWH-133 drives microglia toward an anti-inflammatory state.

*In vivo*, we administered a concentration of JWH-133 (1mg/kg) in line with different models of brain injuries (Murikinati et al., 2010; Gamal et al., 2015; Fujii et al., 2016; Cakir et al., 2019). As previously described, microglia serve as the primary immune cells of the brain, releasing various pro-inflammatory cytokines (Hanisch, 2002). We were able to determine differences in microglia morphology by measuring endpoints per cell and branch length per cell. These measurements are relevant because previous studies have shown that activation states of microglia cells are associated with branching (Davis et al., 1994; Karperien et al., 2013; Gomez-Nicola and Perry, 2015). Activated microglia possess a round amoeboid shape, with little to no branching. These characteristics are indicative of “active” microglia, which function as macrophages in response to injury, and secrete several pro-inflammatory cytokines like IL-1, IL-6, and TNF (Davis et al., 1994; Smith et al., 2012; Davis et al., 2017). Pro-inflammatory functions of active microglia have been shown to directly contribute to neuronal death and neurodegeneration (Vezzani et al., 1999; Takeuchi et al., 2006). Thus, upon infection, we expect microglia to have significantly fewer endpoints per cell and smaller branch lengths than in healthy animals. After infection with *S*. *pneumoniae* and treatment with CRO, Iba-1+ cells indeed possessed significantly fewer endpoints and smaller branch lengths than other experimental groups. Due to the bacteriolytic and pro-inflammatory nature of antibiotics like CRO (Grandgirard et al., 2007b; Muri et al., 2018), the increase in microglia activation could be the result of both bacterial infection and antibiotic treatment. Following adjuvant treatment with JWH-133, the Iba-1+ cells demonstrated significantly increased endpoints and branch lengths, suggesting modulation towards a resting and/or intermediate hypertrophied state. In contrast, JWH-133 was not able to influence overall microglial density in the parenchyma. Further, we did not observe differences between uninfected and infected animals. A significant difference upon infection was demonstrated by Dörr and colleagues in an experimental model of pneumococcal meningitis in mice (Dorr et al., 2015). However, their analysis focused on the hippocampal formation, which was not the case in our study. In conjunction with data from *in vitro* glial cells, our analysis suggests that JWH-133 is effective in modulating microglia phenotype away from a reactive state with phagocytotic activity, and through this pathway, reduces neuroinflammation in PM.

The acute phase of PM is characterized by significantly increased levels of inflammatory cytokines released by resident brain immune cells and infiltrating leukocytes (Mook-Kanamori et al., 2011). We measured cytokine levels in the brain parenchyma of infected animals to further understand how adjuvant JWH-133 treatment modulates neuro-inflammation. Our results showed that at 24 and 42 hpi, adjuvant JWH-133 therapy significantly reduced neutrophil chemoattractants CINC-1, CINC-2α/β, and CINC-3. Massive neutrophils infiltration to the CNS is observed in acute PM, forming neutrophil extracellular traps (NETs) in the CSF, which have been shown to trap pneumococcal bacteria and hinder bacterial clearance (Mohanty et al., 2019). Invading leukocytes act also as sources of pro-inflammatory mediators, such as reactive oxygen species and matrix-metalloproteinases (Meli et al., 2003). In these cells, CB2 activation also result in anti-inflammatory effects (Murikinati et al., 2010; Kapellos et al., 2019). The specific involvement of neutrophils was not the primary focus of the present study. The reduction in the production of chemoattractants in brain parenchyma by JWH-133 may attenuate the recruitment of neutrophils. This has been proposed as a mechanism for JWH-133-mediated attenuation of brain damage following ischemia in rats subjected to middle cerebral artery occlusion (MCAO) (Murikinati et al., 2010). This would merit further investigation in our experimental model. In addition, JWH-133 also down-regulates MIP-1a and MIP-3α in our model. Macrophage inflammatory proteins are secreted by brain-resident macrophages, and have been shown to be up-regulated in pneumococcal meningitis, where they are involved in the recruitment of leukocytes into the CNS (Driscoll, 1994; Coutinho et al., 2013). Pro-inflammatory interleukins such as IL-6 and IL-1α were also down-regulated by JWH-133 administration. Microglia function has been described as one of the primary mediators of immune response in the brain following infection (Barichello et al., 2016; Thorsdottir et al., 2019), and the attenuation of inflammatory responses in brain tissue following administration of JWH-133 further corroborates that JWH-133 may be a promising neuro-protective therapy targeting microglial cells.

Acute inflammation in the subarachnoid space and the ensuing vasculitis, as well as cerebral thrombosis, are believed to be the causes of cortical damage in PM (Leib et al., 1996; Nau and Bruck, 2002). Adjuvant JWH-133 therapy didn’t reduce brain damage in rats infected with PM. This is in contrast to the neuroprotective effect of JWH-133 observed in experimental models of okadaic acid-induced neuroinflammation and damage (Cakir et al., 2019) or MCAO-induced ischemia (Murikinati et al., 2010). However, in these models, JWH-133 was applied at the time or shortly before surgery. In our model, JWH-133 was given as adjuvant therapy to antibiotics, a paradigm more relevant to clinical application. This may explain the lack of effect observed on brain damage, although the anti-inflammatory effect on microglia is demonstrated. In previous studies from our group, decrease in inflammatory parameters by different adjunctive therapies was associated with improved neuroprotection and attenuation of cortical damage (Grandgirard et al., 2007b; Liechti et al., 2014; Muri et al., 2018). In particular, the attenuation of cortical damage was consistently associated with a reduction of MMP-9 activity (Liechti et al., 2015). In the present study, the expression of parenchymal MMP-9 was not reduced by JWH-133, in line with its inability to decrease cortical damage.

JWH-133 therapy was shown to improve outcome in term of neurofunctional behavior in different experimental models. In particular, it attenuated the impairment of Morris watermaze performance induced by okadaic acid treatment in rats (Cakir et al., 2019). In the present study, we only focused on the acute phase of the disease and didn’t investigate a possible effect of the chronic application of JWH-133 on later neurofunctional parameters. Such a study was performed on adult rats with pneumococcal meningitis using cannabidiol for treatment (Barichello et al., 2012). A reduction in the host immune response and a prevention of cognitive impairments were documented. However, therapy was initiated at the time of infection. Further, in contrast to JWH-133, the effects of CBD are very unspecific, which makes a direct comparison between the two treatments subject to caution. Specific endocannabinoid modulation of microglia drives their polarization toward a phenotype warranting therapeutic functionality, with not only anti-inflammatory and neuroprotective effect, but also tissue-remodeling or regenerative capacity. This is the fundament proposed for the treatment of different neuropathologies by endocannabinoids (Tanaka et al., 2020). Cannabinoids are potent regulators of neural stem cell (NSC) biology (Rodrigues et al., 2019). JWH-133 has been shown to increase NSC proliferation in the subventricular zone (Goncalves et al., 2008), and another CB2 agonist, AM1241, enhanced cell proliferation in the hippocampus of mice displaying deficits in neurogenesis (Avraham et al., 2014). Based on these observations, chronic application of JWH-133 during experimental pneumococcal may hold more potential to support regeneration and improve the neurofunctional outcome of infected animals than influencing the acute reaction, when used in a clinically relevant therapeutic modality.

Our study has several limitations: 1) by using a primary mixed glial culture, the effect of JWH-133 was not specifically investigated on microglia, but more on a general glial population consisting of astrocytes and microglia. Astrocytes are likely to also be regulated by CB2 agonists (Kozela et al., 2017). This could however better reflect the *in vivo* situation in the parenchyma. 2) Neutrophils participate in the hyperinflammatory state during pneumococcal meningitis. Unfortunately, the effect of JWH-133 treatment on the recruitment of these cells in the CSF couldn’t be determined, given the limited quantity of CSF that could be harvested from infant rats.

In conclusion, we could demonstrate both *in vitro* and *in vivo* the ability of JWH-133 to modulate microglial behavior to a non-inflammatory phenotype. When applied as adjuvant therapy, this was however not effective in improving clinical outcome and brain damage in the acute phase. Given the proven effect on microglia and its potential to support neuronal regeneration, JWH-133 may hold promise in a chronic application.

## Data Availability Statement

The raw data supporting the conclusions of this article will be made available by the authors, without undue reservation.

## Ethics Statement

The animal study was reviewed and approved by Animal Care and Experimentation Committee of the Canton of Bern, Switzerland (license no. BE 1/18).

## Author Contributions

SDP performed the experiments, analyzed the results, and contributed to study design and manuscript preparation. DG contributed to study design, supervised the experiments, analyzed the results, and contributed to manuscript preparation. SLL supervised the experiments, participated in the study design, and contributed to manuscript preparation. All authors contributed to the article and approved the submitted version.

## Funding

The work was supported by a grant from the Swiss National Science Foundation (no. 189136) to SLL and a Swiss Government Excellence Scholarship (0532) awarded to SDP.

## Conflict of Interest

The authors declare that the research was conducted in the absence of any commercial or financial relationships that could be construed as a potential conflict of interest
