# Supplementary material for: Adjuvant Cannabinoid Receptor Type 2 Agonist Modulates the Polarization of Microglia Towards a Non-Inflammatory Phenotype in Experimental Pneumococcal Meningitis
Source: Front Cell Infect Microbiol. 2020 Nov 5;10:588195. doi: 10.3389/fcimb.2020.588195 (PMC7674855; doi:10.3389/fcimb.2020.588195)
Supplement: Supplementary file 1 [file DataSheet_1.pdf]

Supplementary Material

1 Supplementary Materials and Methods

1.1 Gelatin-sepharose affinity binding

Gelatinases were enriched by incubating 300 µg of brain homogenate with 20 µl of Gelatin Sepharose 4B (GE Healthcare GmbH, Glattbrugg, Switzerland) on an orbital shaker overnight at 4°C. Sepharose beads were washed (50 mM Tris-HCl pH 7.6, 150 mM NaCl, 5 mM CaCl2, 0.02% NaN3, 0.05% Brij 25, 1% Triton X-100) and incubated with 2X zymography sample buffer (30 mM Tris HCl pH 6.8, 20% glycerol, 1% sodium dodecyl sulphate (SDS), 0.02% bromophenol blue) for 5 min at room temperature to elute bound proteins. After centrifugation, supernatants were collected and tested by gelatin gel zymography.

1.2 Gelatin zymography

Gelatin-sepharose-purified samples were subjected to electrophoresis under non-reducing conditions in polyacrylamide gels containing 1% (v/v) type A gelatin from porcine skin (Sigma-Aldrich). After electrophoresis, SDS was removed from the gels, and the MMP catalytic sites were activated by overnight incubation at 37oC in zymography buffer (10 mM CaCl2, 50 mM Tris, 50 mM NaCl, pH 7.65). Gels were scanned and gelatinolytic activities (MMP-9, gelatinase B, 92 kDa and MMP-2, gelatinase A, 72 kDa) were determined by quantification of gelatin lysis zones using Image J software and expressed as absolute numbers for each sample. Thereof, relative values of MMP-9/MMP-2 ratios were calculated as MMP-2 is constitutively expressed.

2 Supplementary Figures

2.1 Supplementary Figure 1

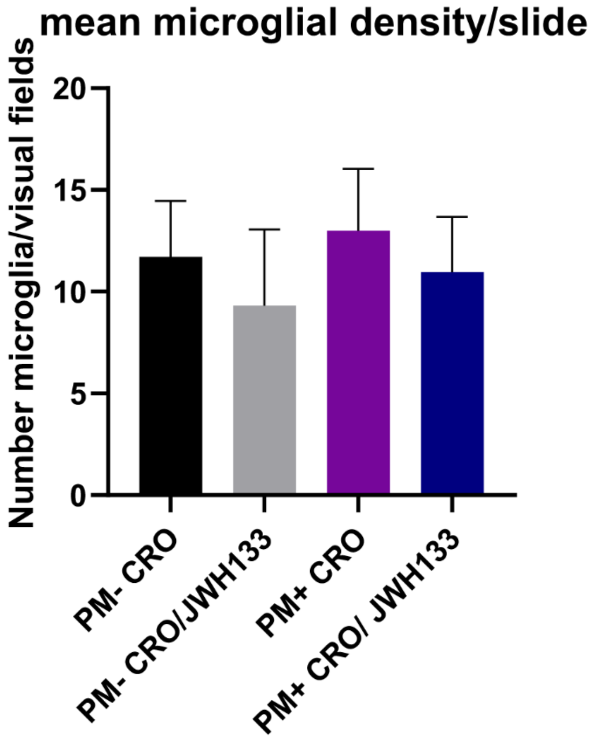

Supplementary Figure 1: Quantitative assessment of microglial density on Iba-1 immuno-stained sections: Neither infection, nor treatment significantly influenced the total number of microglia counted per analyzed image (Two-way ANOVA) (PM- CRO n=3; PM- CRO/JWH133 n=4; PM+ CRO n=8; PM+ CRO/JWH133 n=4).

2.2 Supplementary Figure 2

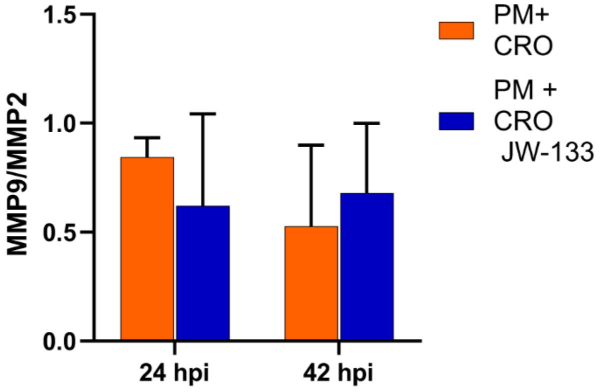

Supplementary Figure 2 Level of MMP-9 in brain homogenates determined by gelatin gel zymography. No significant differences could be detected between infected animals treated with CRO alone (orange bars) or CRO/JHW133 (blue bars) at 24 hpi (PM + CRO n=3, PM+ CRO/JWH133 n=3; p=0.67), or at 42 hpi (PM + CRO n=6, PM+ CRO/JWH133 n=6; p=0.69) (2 way ANOVA with Sidak correction).
